# High Human Papillomavirus Vaccine Acceptability and Cost-Effectiveness of the Chinese 2-Valent Vaccine Among Men Who Have Sex With Men: A Cross-Sectional Study in Shenyang, China

**DOI:** 10.3389/fmed.2021.763564

**Published:** 2021-11-19

**Authors:** Ze-Hao Ye, Zhao-Zhen Liu, Si-Tong Cui, Zhen-Xing Chu, Yong-Jun Jiang, Jun-Jie Xu, Qing-Hai Hu, Hong Shang

**Affiliations:** ^1^National Health Commission Key Laboratory of AIDS Immunology (China Medical University), National Clinical Research Center for Laboratory Medicine, The First Affiliated Hospital of China Medical University, Shenyang, China; ^2^Key Laboratory of AIDS Immunology, Chinese Academy of Medical Sciences, Shenyang, China; ^3^Key Laboratory of AIDS Immunology of Liaoning Province, Shenyang, China; ^4^Collaborative Innovation Center for Diagnosis and Treatment of Infectious Diseases, Hangzhou, China

**Keywords:** human papillomavirus, vaccine, men who have sex with men, acceptability, cost-effectiveness, China

## Abstract

**Background:** Despite the insupportable burden caused by the human papillomavirus (HPV) and high vaccine acceptability, vaccination programs are not currently available for men who have sex with men (MSM). We aimed to assess HPV infection by examining the willingness for vaccination among MSM and cost-effectiveness of the Chinese 2-valent HPV vaccine.

**Methods:** We recruited MSM in Shenyang, China between July and December 2020 to conduct anal HPV testing and an online survey regarding HPV-related knowledge and vaccine acceptability. We performed a cost-effectiveness analysis to evaluate the incremental cost-effectiveness ratios (ICERs) of the Chinese 2-valent HPV vaccine.

**Results:** A total of 234 participants completed the online survey; of those, 203 were successfully tested for HPV. The median age was 30 years [interquartile range (IQR): 23–38 years]. Most participants had at least undergraduate education (136/234, 58.1%). The acceptability rate for the free HPV vaccine was 57.7% (135/234). The prevalence of HPV types 16 and 18 was 14.9% (18/121) and 26.8% (22/82) in the willing and unwilling to vaccinate groups, respectively (*P* > 0.05). The prevalence of high-risk HPV among participants aged <30 and ≥50 years was 48.6 and 38.9%, respectively. Using the Chinese per capita gross domestic product (GDP) as a threshold, the Chinese 2-valent HPV vaccine would be a “very cost-effective” strategy, with an ICER value of USD 4,411. This evidence showed that the Chinese 2-valent HPV vaccine was more cost-effective than other imported vaccines.

**Conclusions:** Targeted strategies should be utilized in MSM with different rates of vaccine acceptability. A pilot HPV vaccination program based on the Chinese 2-valent HPV vaccine for MSM is urgently warranted to reduce the burden of HPV and anal cancer.

## Introduction

Men who have sex with men (MSM) are at a higher risk of infection with human papillomavirus (HPV) than females and heterosexual males with a global prevalence of 53.6 and 89.0% among human immunodeficiency virus-negative (HIV-negative) and HIV-positive MSM, respectively (1, 2). The incidence of anal cancer caused by HPV in HIV-negative MSM even rival to that of cervical cancer in females (17/100,000 vs. 13/100,000 person years) (3, 4). In China, MSM are suffering a similar burden caused by HPV and anal cancer (5).

Considering the heavy burden caused by HPV, there is high acceptability of vaccination against HPV among MSM. According to a recent systematic review, approximately half of MSM is willing to receive an HPV vaccine (6). A multicentric study showed that, in China, this rate was 67.5% (7). However, previous studies have only separately examined infection with HPV or vaccine acceptability among MSM (7–11). Further investigation of the relationship between infection with HPV and willingness to receive a vaccine is warranted to determine the benefits of vaccination in MSM.

Despite the insupportable burden caused by HPV and strong willingness to effectively prevent infection with HPV, MSM are not included in the HPV prevention programs of most countries. Three licensed HPV vaccines (i.e., 2-, 4-, and 9-valent) are currently available to prevent HPV-related disease (12) and have been shown to be effective in MSM (13). However, only a small number of high-income countries, such as the United States of America, Australia, and Canada, have extended vaccine recommendations to MSM (14), failing to meet the great demand in this population (7, 15, 16). Given the limited vaccine production and high cost, a gender-neutral HPV vaccination strategy, which is highly effective in eradicating oncogenic infection with HPV (17), has been proposed in debates (18). For similar reasons, MSM have not yet been included in HPV vaccination programs in mainland China, except for Hongkong, China (19). In 2019, the Chinese Food and Drug Administration approved the first Chinese 2-valent HPV vaccine (Cecolin®, Innovax, Xiamen, China). Based on its considerably lower cost, its development may provide an opportunity to extend recommendations for vaccination to MSM. A cost-effectiveness analysis of the Chinese 2-valent HPV vaccine among MSM is required. Such an analysis would assist public health policymakers in evaluating the feasibility of including this population into vaccination programs against HPV.

We sought to assess infection with HPV based on the different rates of willingness for vaccination among MSM and cost-effectiveness of the Chinese 2-valent HPV vaccine.

## Methods

### Study Design and Participant Enrolment

We conducted a cross-sectional study between July and December 2020 to investigate the acceptability of the HPV vaccine and anal infection with HPV among MSM. This study was conducted through a voluntary counseling and testing clinic in The First Affiliated Hospital of China Medical University in Shenyang, China. Participants aged ≥18 years and willing to participate in the study were enrolled. All recruited participants completed an online questionnaire (duration: approximately 10 min) and, subsequently, anal specimens were collected. All participants were compensated with 50 RMB (approximately USD 7.77) for their time. In this study, we utilized the Checklist for Reporting Results of Cross-sectional Studies (see Supplementary Material 1).

### Data Collection

After receiving information regarding the content and protocol of the study, participants completed a self-administered online questionnaire on sociodemographic information (i.e., age, sex, educational background, working status, marital status, and monthly income), sexual behavioral characteristics, HPV-related knowledge, HPV vaccine acceptability, and use of pre-exposure prophylaxis (PrEP) for HIV (questionnaire in English version is shown in Supplementary Material 2). Sexual behavioral characteristics included sex role, number of male partners, number of insertive male partners, condom use, and substance use. The five questions on HPV-related knowledge were as follows: (1) Which is the main transmission route of HPV among men?; (2) Which type of cancer is caused by HPV among women?; (3) Which type of cancer is caused by HPV among men?; (4) Which is the main method for HPV testing in men?; and (5) Could HPV cause condyloma acuminata in both men and women? Each internet protocol address was restricted to ensure that each participant completed the questionnaire only once.

### Collection of Anal Specimens and HPV Testing

Anal specimens were collected by a skilled sampling technician for HPV testing. During the process of anal swab sampling, the elbow-knee position was adopted. The technician inserted a swab into the anal canal (depth: 5 cm), which was rotated left and right for 20 s. For consistency, a single technician collected all anal specimens. Collected specimens were stored in PreservCyt and at −20°C before transportation to Kaipu Medical Laboratory in Shenyang, China for polymerase chain reaction analysis and genotyping for the 23 types of HPV. These types of HPV were classified as high-risk HPV (HR-HPV) and low-risk HPV (LR-HPV). High-risk HPV are types 16, 18, 31, 33, 35, 39, 45, 51, 52, 56, 58, 59, 66, and 68; LR-HPV are types 6, 11, 42, 43, 44, 53, 73, 81, and 82. The 2-valent vaccine covers HPV types 16 and 18; the 4-valent vaccine covers HPV types 6, 11, 16, and 18; and the 9-valent vaccine covers HPV types 6, 11, 16, 18, 31, 33, 45, 52, and 58.

### Sample Size Calculation

We calculated the sample size using the Power Analysis and Sample Size (PASS) Version 15.0 software (NCSS, LLC, Kaysville, Utah, USA) based on a two-sided alpha of 0.05 and power of 0.8. Based on the results of previous studies on HPV vaccine acceptability among MSM, we set the estimated acceptability rate at 60%. The minimal sample size calculated with sufficient power was 194 participants. Considering the estimated 10% non-response rate, we calculated the expected sample size to be 214 participants.

### Cost-Effectiveness Analysis

The cost-effectiveness analysis was reported according to the ISPOR Health Economic Evaluation Publication Guidelines-CHEERS Checklist and the HPV-FRAME reporting standards (see Supplementary Materials 3, 4, respectively). We analyzed four HPV vaccination scenarios, i.e., vaccinations with 2-valent (Cecolin®, Innovax, Xiamen, China), 2-valent (Cervarix®), 4-valent (Gardasil®), and 9-valent (Gardasil 9®) vaccine. We calculated the gained quality-adjusted life-years (QALYs) by comparing two groups willing and non-willing to receive the vaccine. The cost-effectiveness of vaccines was measured by the incremental cost-effectiveness ratio (ICER), the ratio of extra costs from vaccination divided by the QALYs gained. Based on the definition of cost-effectiveness established by the World Health Organization (20), strategies with an ICER less than China's per capita gross domestic product (GDP) in 2020 (USD 10,484) (21) and less than three times China's per capita GDP (USD 31,452) were considered “very cost-effective” and “cost-effective,” respectively. We also conducted one-way and multi-way sensitivity analyses for a robust evaluation of cost-effectiveness. All costs were reported in 2020 USD and discounted at a discount rate of 3%. Details are provided in Supplementary Material 5.

### Statistical Analysis

For variables with a missing ratio <5% in the course of data processing, we imputed missing values using the mean and mode for continuous and categorical variables, respectively. In this study, there were no variables with a missing ratio >5%, which they would have been deleted. We used proportions for categorical variables, such as knowledge regarding HPV and vaccine acceptability. We used median and interquartile range (IQR) for the age of participants. We computed a score of HPV-related knowledge (range: 0–5) based on the above five questions (correct = 1, wrong or not know = 0). Knowledge regarding HPV was categorized into low (0), middle (1–3), and high (4, 5) levels to evaluate its impact on HIV vaccine acceptability. We performed binary logistic regression analysis to examine the association between potential factors and acceptability of HPV vaccination. In the multivariable logistic regression analysis, we adjusted age, educational background, working status, marital status, and monthly income to calculate adjusted odds ratios (aOR) and their 95% confidence intervals (CIs). All statistical analyses were performed with SPSS Statistics version 26.0 (IBM Corporation, Armonk, NY, USA). Variables with two-tailed *P*-values <0.05 were considered statistically significant.

## Results

### Characteristics of Participants

**Figure 1:**
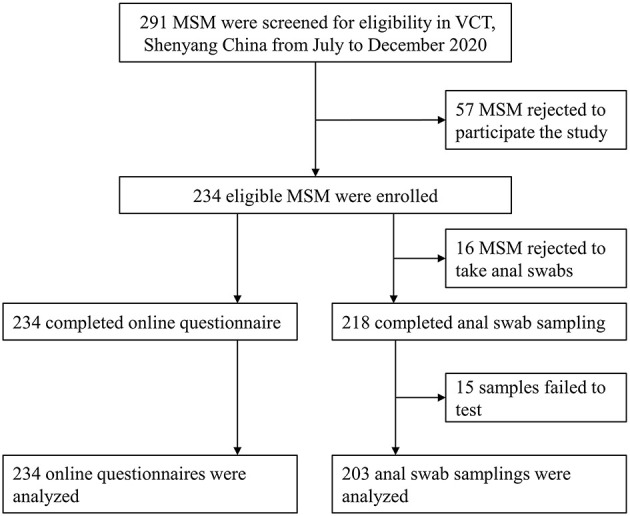
Flow chart. MSM, men who have sex with men; VCT, voluntary counseling and testing.

Of note, 80.4% (234/291) of all eligible participants were included in this study. Among those, 93.2% (218/234) completed anal specimen collection and 86.8% (203/234) had available HPV testing results (Figure 1). The median age of participants was 30 years (IQR: 23–38 years). Most participants had at least undergraduate education (136/234, 58.1%), were single (149/234, 63.7%), and had a monthly income ranging RMB 2,000–4,999 (115/234, 49.1%). Also, 65 participants (29.6%) were using PrEP for HIV (Table 1).

**Table 1:** Demographic, sexual behavioral characteristics, knowledge regarding HPV, and STI testing results among 234 unvaccinated MSM.

	***N***	**%**
**Age (median, IQR)**	30	23–38
18–29	115	49.1
30–39	70	29.9
40–49	25	10.7
>50	24	10.3
**Educational background**		
Senior high school or below	98	41.9
Undergraduate or above	136	58.1
**Working status**		
Full-time	117	50.0
Others	117	50.0
**Marital status**		
Single	149	63.7
Married	27	11.5
Others	58	24.8
**Monthly income (RMB)**		
<2,000	58	24.8
2,000–4,999	115	49.1
>5,000	61	26.1
**Sex role in the past 6 months**		
Both	54	23.1
Only insertive	94	40.2
Only receptive	86	36.8
**Number of male sexual partners in the past 6 months**		
None	22	9.4
1–2	125	53.4
≥3	87	37.2
**Condom use with male sexual partners in the past 6 months**		
Each time	134	57.3
Other	100	42.7
**Having sex with females in the past 6 months**		
No	201	85.9
Yes	33	14.1
Substance use during sexual intercourse in the past 6 months*		
No	161	69.4
Yes	71	30.6
**History of STIs**		
No	181	77.4
Yes	53	22.6
**HPV knowledge score**		
0	105	44.9
1–3	89	38.0
4–5	40	17.1
**Willingness to receive free HPV vaccination**		
Yes	135	57.7
Other^a^	99	42.3
**Using PrEP for HIV** ^**#**^		
No	155	70.5
Yes	65	29.5
**HPV-positive in the anal sample** ^**#**^		
No	82	40.4
Yes	121	59.6
**HR-HPV in the anal sample** ^**#**^		
No	104	51.2
Yes	99	48.8
**HIV-positive** ^**#**^		
No	200	90.5
Yes	21	9.5

*HIV, human immunodeficiency virus; HPV, human papillomavirus; HR-HPV, high-risk-HPV; MSM, men who have sex with men; IQR, interquartile range; PrEP, pre-exposure prophylaxis; STI, sexual transmission infection*.

# *Counts of these variables are not equal to 234 due to missing data*.

a *Others include respondence “No” (24) and “I don't know” (75)*.

### Sexual Behavior, Knowledge Regarding HPV, and Vaccine Acceptability

In the past 6 months, most participants played an only-insertive sex role (94/234, 40.2%) and had 1–2 male sex partners (125/234, 53.4%). Furthermore, 33 participants (14.1%) had female partners, and 71 participants (30.6%) self-reported that they used substances during or before sexual intercourse in the past 6 months (Table 1).

Table 1 also shows data on HPV-related knowledge and vaccine acceptability. Only 40 participants (17.1%) had a score of 4–5 with regard to HPV-related knowledge; 105 participants (44.9%) did not know the correct answer of all five questions. In total, 135 participants (57.7%) self-reported that they would like to receive the free HPV vaccine if it was available for them.

### Anal Infection With HPV Among Participants With Different Degrees of Willingness for HPV Vaccination

**Figure 2:**
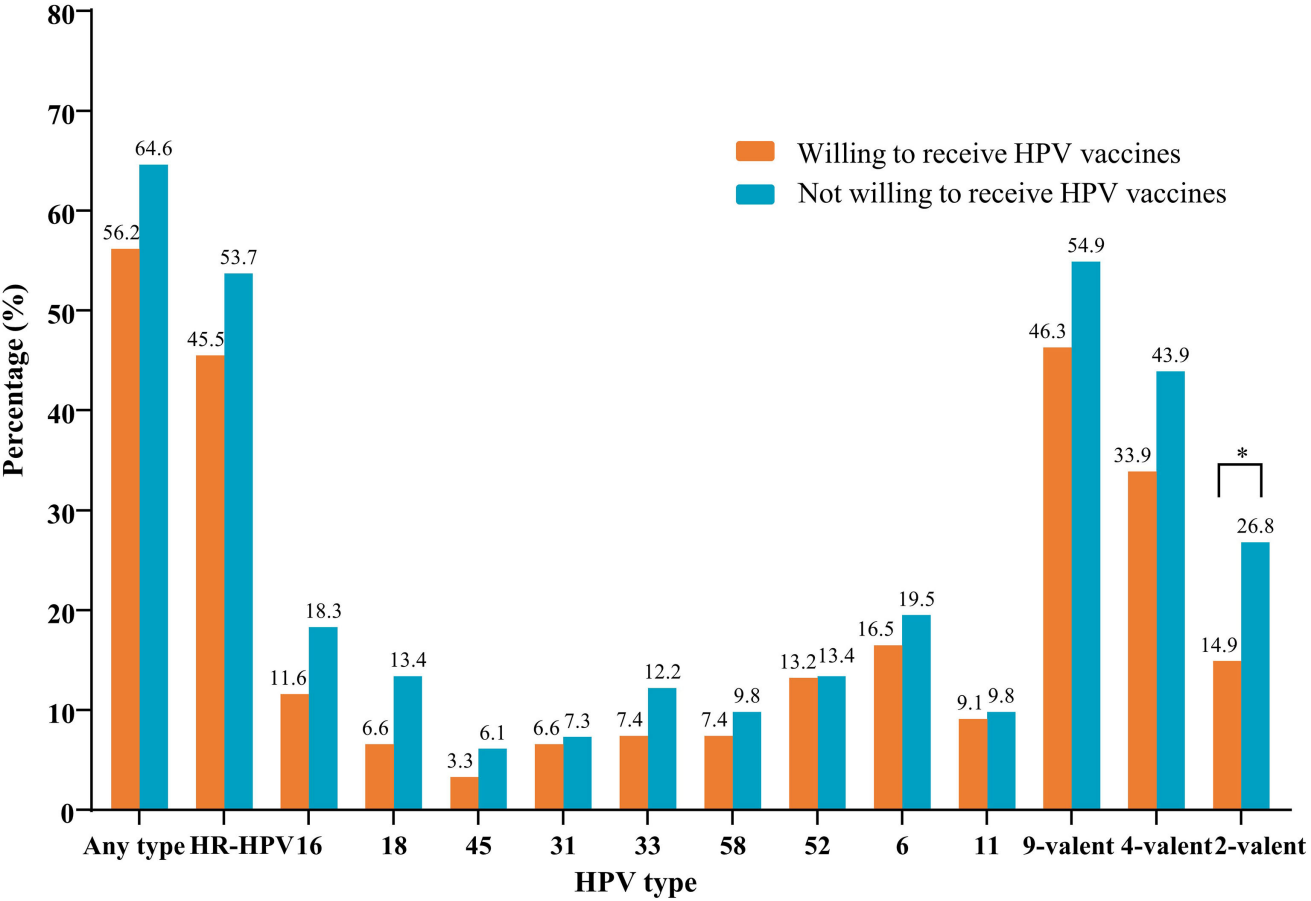
Prevalence of nine HR-HPV types stratified by the willingness of 203 MSM to receive vaccines. HPV types 16 and 18 were prevented by the 2-valent vaccine; HPV types 16, 18, 6, and 11 were prevented by the 4-valent vaccine; HPV types 16, 18, 45, 31, 33, 58, 52, 6, and 11 were prevented by the 9-valent vaccine. *P < 0.05. HPV, human papillomavirus; HR-HPV, high-risk-HPV; MSM, men who have sex with men.

The types of HPV were tested in a total of 218 anal swab samples; of those, 15 were excluded due to unsatisfactory sampling. The overall prevalence of anal HPV and HR-HPV was 59.6% (121/203) and 48.8% (99/203), respectively. Figure 2 displays the prevalence of anal HR-HPV and HPV types prevented by the 9-valent vaccine, stratified by the willingness for HPV vaccination among 203 MSM who were successfully tested for HPV. In the group of participants willing to receive a vaccine, the prevalence of anal infections with HPV of any type, high-risk types, and the 2-valent, 4-valent, and 9-valent vaccine types was 56.2, 45.5, 14.9, 33.9, and 46.3%, respectively. In the group of participants who were unwilling to receive a vaccine, these rates were 64.6, 53.7, 26.8, 43.9, and 54.9%, respectively. We observed a significant difference in anal infection with the 2-valent vaccine types of HPV between the two aforementioned groups (14.9 vs. 26.8%, respectively, *P* < 0.05).

**Figure 3:**
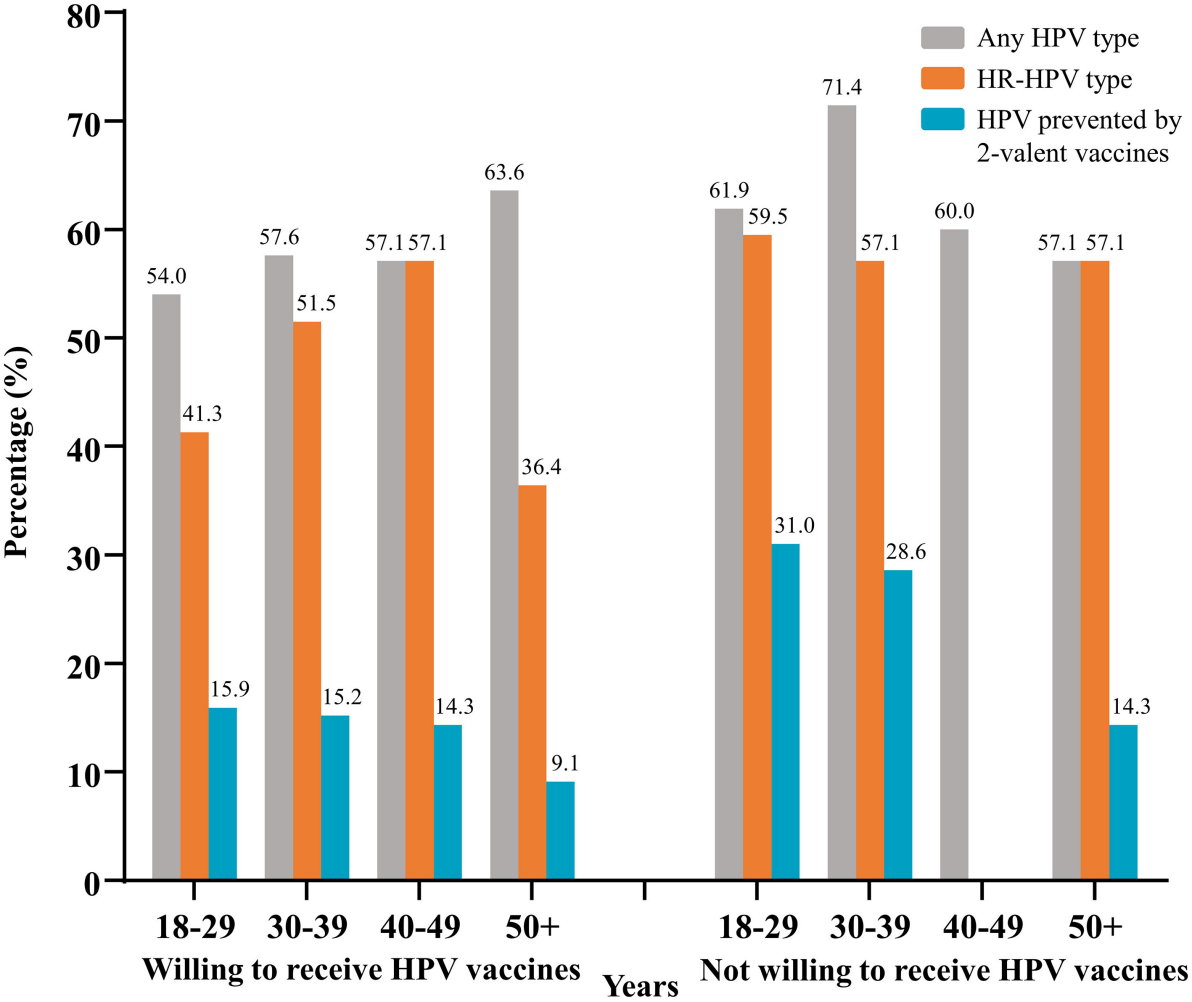
Prevalence of HPV from different age groups stratified by the willingness to receive vaccines. Data was from 203 MSM successfully tested for HPV. HPV, human papillomavirus; HR-HPV, high-risk-HPV; MSM, men who have sex with men.

We found a high prevalence of HR-HPV across all age subgroups of 203 MSM, ranging from 38.9 to 54.1% (data not shown). In the group of participants willing to receive a vaccine, the prevalence of HR-HPV was 41.3, 51.5, 57.1, and 36.4%, for MSM aged 18–29, 30–39, 40–49, and >50 years, respectively. Relatively higher prevalence of HR-HPV was observed in the group of participants not willing to receive a vaccine. No significant difference was found among the different age groups (Figure 3).

### Factors Associated With Acceptability of the Vaccine Against HPV and Infection With HR-HPV

**Figure 4:**
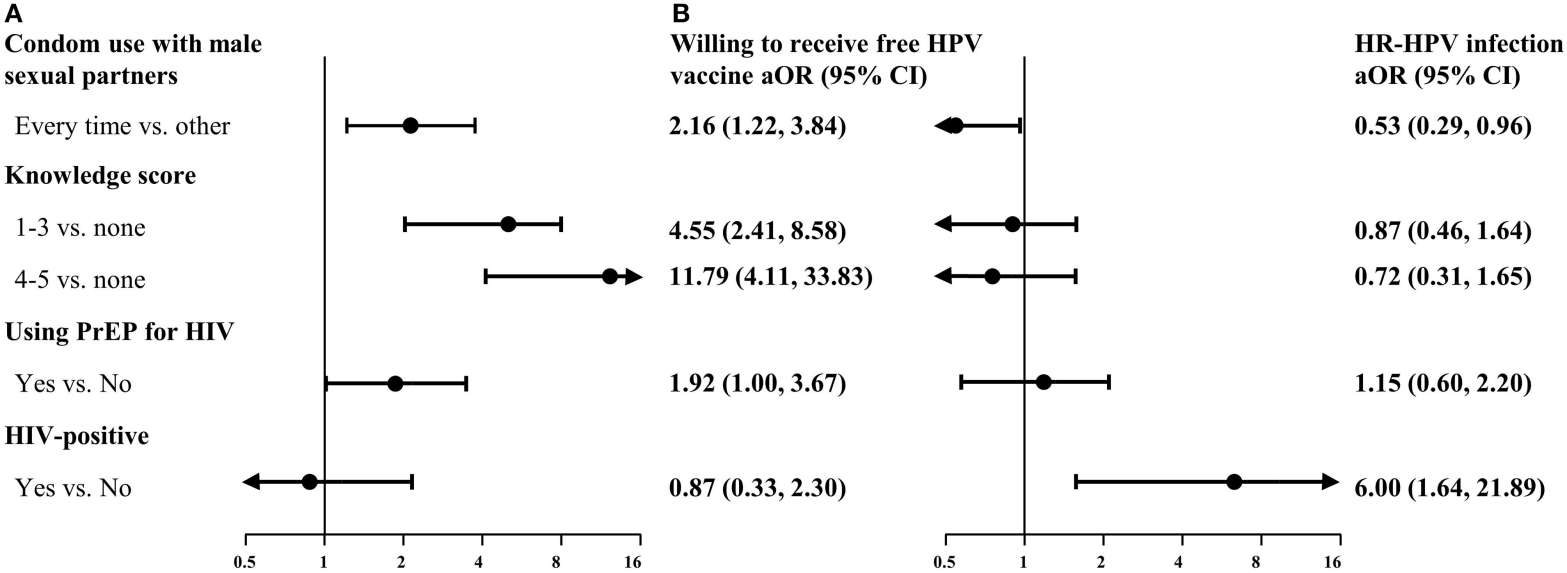
Multivariate analysis of factors associated with willingness to receive free HPV vaccines **(A)** and HR-HPV infection **(B)**. Data in **(A)** and **(B)** were from 234 unvaccinated MSM and 203 MSM successfully tested for HPV, respectively. aOR, adjusted odds ratios (obtained after adjustment for age, educational background, working status, marital status, and monthly income); CI, confidence interval; HIV, human immunodeficiency virus; HPV, human papillomavirus; HR-HPV, high-risk-HPV; MSM, men who have sex with men; PrEP, pre-exposure prophylaxis.

Figure 4 shows the odds ratios for participants willing to receive the free HPV vaccine and those infected with HR-HPV, adjusted for age, educational background, working status, marital status, and monthly income. Potential facilitators associated with willingness to receive the free HPV vaccine among MSM included the use of condoms with male sexual partners in the past 6 months (aOR: 2.16; 95% CI: 1.22–3.84), a high score for knowledge regarding HPV (aOR: 11.79; 95% CI: 4.11–33.83), and use of PrEP for HIV (aOR: 1.92; 95% CI: 1.00–3.67). Human immunodeficiency virus-positive MSM were at increased risk of infection with HR-HPV (aOR: 6.00; 95% CI: 1.64–21.89). Condom use with male sexual partners in the past 6 months (aOR: 0.53; 95% CI: 0.29–0.96) had a significantly negative association with HR-HPV infection.

### Cost-Effectiveness Analysis

The costs of four vaccination scenarios (three doses), namely 2-valent (Cecolin®), 2-valent (Cervarix®), 4-valent (Gardasil®), and 9-valent (Gardasil 9®) vaccines, were USD 18,595, USD 34,194, USD 46,499, and USD 74,749, respectively. According to China's per capita GDP in 2020, the 2-valent (Cecolin®) vaccination strategy was “very cost-effective.” Compared with those of other scenarios, the 2-valent (Cecolin®) vaccination scenario was more cost-effective (ICER: 4,411 vs. 7,929, 10,614, and 14,217, respectively; Table 2). The ICER for the 2-valent (Cecolin®) vaccine was consistently lower than those of other scenarios in both one-way (from USD 2,328 to USD 8,739) and multi-way (from USD 1,369 to USD 21,384) sensitivity analyses; therefore, this scenario was considered at least “cost-effective” (see details in Supplementary Material 5).

**Table 2:** Evaluation of the cost-effectiveness of different HPV vaccination scenarios using samples collected from 203 MSM.

**Scenarios**	**2-valent (Cecolin^®^) vaccine**	**2-valent (Cervarix^®^) vaccine**	**4-valent (Gardasil^®^) vaccine**	**9-valent (Gardasil 9^®^) vaccine**
Vaccination cost (USD)	26,432	42,031	54,337	82,587
Direct medical cost averted (USD)	6,876	6,876	6,876	7,685
Incremental cost (USD)	19,557	35,156	47,461	74,902
Number of QALYs gained (year)	4.43	4.43	4.43	5.27
ICER (USD per QALY)	4,411	7,929	10,705	14,217

*Vaccination costs included those incurred from HPV testing, vaccine, storage, transportation, and vaccination service. HPV, human papillomavirus; ICER, incremental cost-effectiveness ratio; MSM, men who have sex with men; QALY, quality-adjusted life-year*.

## Discussion

### Principal Findings and Significance

Coinciding with the increasing willingness for HPV vaccination among MSM in China and the release of the first locally produced HPV vaccine in 2019, which is associated with lower costs compared with imported vaccines, we analyzed the prevalence of infection with HPV in combination with the willingness for vaccination among MSM and cost-effectiveness of the Chinese 2-valent HPV vaccine. We found that most participants, of whom nearly half had been infected with HR-HPV, were willing to receive free HPV vaccines. On-demand administration of the HPV 2-valent (Cecolin®) vaccine may be the most cost-effective strategy for preventing the development of anal cancer among MSM. This study provides additional evidence on the infection with HPV, demand for vaccination against HPV, and cost-effectiveness of the Chinese 2-valent HPV vaccine among MSM. This evidence presents the urgent need for a national pilot HPV vaccination program tailored to MSM. In addition, our results also provide a vital reference to most countries that have not launched HPV vaccination programs for MSM, particularly those with limited resources. The more economical Chinese 2-valent HPV vaccine may also provide another option for gender-neutral vaccination (18).

### Infection With HR-HPV and Vaccine Acceptability

The present findings revealed that the prevalence of HR-HPV among MSM in Shenyang, China was relatively higher (48.8%) compared with that reported in previous studies in Africa (9, 23) and other regions of China (22, 24, 25). However, it was lower than the prevalence observed in Italy (26) and Southeast Asia (27). The high burden of infection with HR-HPV reflects the urgent need for an MSM-targeted HPV vaccination program to break the transmission chain of HPV in this subpopulation. Additionally, similar to other studies (7, 28), we found that MSM had insufficient knowledge regarding HPV. Despite the low levels of knowledge, nearly 60% of MSM were willing to receive free HPV vaccines. This rate is comparable to that (67.5%) recorded in a multicentric study including 10 cities in China (7), but higher than the average rate (50.0%) reported in a 2021 systematic review (6). Nonetheless, vaccine hesitancy (29), having been associated with a lack of knowledge on vaccines, may impede the uptake of HPV vaccines (19). It is warranted to deliver knowledge and correct misinformation on side effects, safety, and effectiveness of HPV vaccines among MSM.

### Anal Infection With HPV Among Participants With Different Degrees of Willingness for Vaccination Against HPV

Previous studies rarely investigated infection with HPV between MSM with different degrees of willingness to receive a vaccine against HPV. Notably, we found that the prevalence of anal HPV (all subgroups) was higher among MSM who were unwilling to receive a vaccine against HPV vs. those willing to receive a vaccine against HPV. The significantly higher prevalence among MSM who are unwilling to receive HPV vaccines was exclusively observed in the 2-valent HPV type subgroup, which may be explained by differences in behavior and awareness regarding protective measures. These findings also suggest that these individuals are at relatively higher risks of anal cancer caused by HPV. At present, MSM-orientated vaccination against HPV is not recommended in China. Therefore, improving the degree of willingness for vaccination against HPV may indirectly assist in avoiding infection with HR-HPV among MSM. Future strategies aimed at improving vaccine uptake and knowledge regarding HPV should focus on the corresponding MSM population to maximize the benefits.

In previous studies, the age-specific prevalence of HPV among MSM was inconsistent. Most investigations reported a stably high prevalence of anal infection with HPV in all age groups (30, 31). The present study yielded similar results, indicating that HPV causes a heavy burden in both young and older MSM. Although HPV-infected MSM will also benefit from preventing anal cancer through HPV vaccination, its preventive effect is compromised vs. uninfected MSM (32). Therefore, prospective HPV education and vaccination programs should target younger MSM before the onset of sexual behavior.

### Factors Associated With Vaccination Against HPV and Infection With HR-HPV

We identified potential facilitators of vaccination against HPV and factors associated with HR-HPV infection among MSM. As a result of education on self-protection and self-care (33), MSM with a high score for knowledge regarding HPV had a significantly higher willingness to receive a vaccine against HPV. This indicates that HPV awareness campaigns and education targeting MSM are necessary to maximize the benefits from vaccination against HPV. MSM who used condoms each time during intercourse and used PrEP for HIV also had a significantly higher willingness to receive a vaccine against HPV compared with those who did not. Previous studies showed that MSM receiving PrEP services are at higher risks for HR-HPV infection (34) and probably have more access to information regarding vaccines against HPV (35). Therefore, future efforts to promote vaccination against HPV among MSM should combine HPV-related education with promotion for the use of condoms and PrEP services. For example, a tailored web-based intervention could be effective in improving the attitude and belief of individuals regarding self-protection (36). We also found that factors influencing infection with HR-HPV included condom use and HIV infection, highlighting the necessity to combine strategies for the prevention of HPV and HIV.

### Cost-Effectiveness Analysis

In this study, we found that the 2-valent (Cecolin®) vaccine was the most cost-effective option for the vaccination of MSM compared with the 2-valent (Cervarix®), 4-valent (Gardasil®), and 9-valent (Gardasil 9®) vaccines. In 2020, the World Health Organization launched the first global strategy to accelerate the elimination of cervical cancer in 194 countries, including China (37). It means more potential resource for HPV prevention will be concentrate on females and that for MSM would be considerably limited for years to come. Given that sexual transmission is the primary spread mode to women, vaccination among men to close the transmission route is hard to negligible on the ambitious process of eliminating cervical cancer. A pilot vaccination program against HPV among MSM should be initiated in China as soon as possible. The lower cost and higher cost-effectiveness of the Chinese 2-valent vaccine facilitate this effort.

## Limitations

There are some limitations in this study. Firstly, selection bias due to the small sample and single enrollment method used in our study may limit the extrapolation of the present findings to the general MSM population. Secondly, a self-administered questionnaire was utilized in the present study; hence, recall bias with regard to characteristics of sexual behavior was inevitable. Thirdly, a causal conclusion on associations cannot be reached due to the cross-sectional trait of this study. Fourthly, given the limitations of our model of cost-effectiveness and the lower efficacy of vaccination among individuals infected with HR-HPV, the range of values for the ICER may not reflect the actual degree of uncertainty. Fifthly, HPV infection among MSM is more common in anal samples compared with oral and genital samples. Therefore, we only currently tested anal samples and correspondingly considered the cost-effectiveness on the prevention of anal cancer, which may underestimate the prevalence of HPV and the cost-effectiveness of vaccines. Thus, a comprehensive analysis based on anal, oral and genital sites results will be conducted in the next step. In addition, this study mainly aimed to appeal HPV vaccine accessibility among MSM in China and did not collect information toward vaccine hesitancy, which is also crucial to the subsequent real uptake of HPV vaccines in this population. Studies on HPV vaccine hesitancy among MSM are warranted in the future. Finally, given the effectiveness and cost after HPV vaccination, the vaccine is only recommended for individuals aged <45 years. However, individuals over 45 years of age also have the right and desire to vaccinate themselves, and may have self-vaccination behavior. Considering the life expectancy (74.7 years old), this population also has relatively long-term necessity of health care. Furthermore, based on the low cost of Chinese bivalent vaccines, we assumed that vaccination strategies are also cost-effective to include this group. Therefore, we included this population in study.

Despite the limitations above, this was the first study on infection with HPV in combination with willingness of individuals for vaccination. The data underscore differentiated strategies for the prevention of HPV among MSM. We also assessed for the first time the cost-effectiveness of the Chinese HPV 2-valent vaccine vs. other imported vaccines among MSM. Furthermore, unlike in previous studies, the vaccine coverage was calculated based on vaccine acceptability using samples rather than estimation, which is closer to the natural condition in this population.

## Conclusions

MSM who are unwilling to receive a vaccine against HPV may suffer a heavier burden caused by HPV types 16 and 18. Therefore, there is a need for strategies aimed at improving knowledge regarding HPV among this population. The Chinese 2-valent vaccine against HPV provides a more economical option for the prevention of anal cancer, particularly in low- and middle-income countries with limited resources. A pilot HPV vaccination program for MSM in China is urgently warranted to reduce the prevalence of HPV and the associated anal cancer burden.

## Data Availability Statement

The raw data supporting the conclusions of this article will be made available by the authors, without undue reservation.

## Ethics Statement

The studies involving human participants were reviewed and approved by the Institutional Review Board Committee of the First Affiliated Hospital of China Medical University in Shenyang, China. The patients/participants provided their written informed consent to participate in this study.

## Author Contributions

Q-HH, J-JX, and HS conceived and designed the research study. Z-HY, Z-ZL, S-TC, Z-XC, and Q-HH collected the data for the study. Z-HY and Q-HH analyzed the data and interpreted the results. Z-HY wrote the first draft of the manuscript. Y-JJ, J-JX, Q-HH, and HS revised the manuscript. All authors have reviewed and approved the final manuscript.

## Funding

This work was funded by the Mega-Projects of National Science Research for the 13th Five-Year Plan (2017ZX10201101) and the National Natural Science Foundation of China (82073620).

## Conflict of Interest

The authors declare that the research was conducted in the absence of any commercial or financial relationships that could be construed as a potential conflict of interest.

## Publisher's Note

All claims expressed in this article are solely those of the authors and do not necessarily represent those of their affiliated organizations, or those of the publisher, the editors and the reviewers. Any product that may be evaluated in this article, or claim that may be made by its manufacturer, is not guaranteed or endorsed by the publisher.
