# Supplementary material for: High Human Papillomavirus Vaccine Acceptability and Cost-Effectiveness of the Chinese 2-Valent Vaccine Among Men Who Have Sex With Men: A Cross-Sectional Study in Shenyang, China
Source: Front Med (Lausanne). 2021 Nov 19;8:763564. doi: 10.3389/fmed.2021.763564 (PMC8639684; doi:10.3389/fmed.2021.763564)
Supplement: Supplementary file 2 [file Data_Sheet_2.PDF]

An online survey on Human Papillomavirus (HPV) vaccine acceptability and associated factors among MSM in Shenyang, China

Thank you for participating in the study of “cross-sectional study on Human Papillomavirus (HPV) vaccine acceptability and associated factors”. For this survey, we would like to know your HPV vaccine acceptability and its influencing factors. This survey is conducted anonymously and your personal information will not be exposed. The information you provide can only be accessed by an authorized investigator from the First Hospital of China Medical University. Findings from the survey will help to guide future policies or research. Please complete this survey truthfully according to the real situation. Thanks for your participation.

Part 1. Demographic characteristics

1_1 What is your date of birth? [fill in the blank] *

____

1_2 What is your ethnicity? [single choice] *

○Han

○Others

1_3 Where is your household registration? [single choice] *

○Rural

○Cities and towns

1_4 What is your education level? [single choice] *

○Primary school

○Junior high school

○Senior high school or technical secondary school

○Undergraduate or Junior college

○Graduate or above

1_5 What is your current work status?? [single choice] *

○Full-time job

○Part-time job

○Unemployed

○Retired

○Others

1_6 What is your marital status? [single choice] *

○Single

○Married

○Cohabitation with male

○Cohabitation with female

○Others

1_7 What is your monthly income?? [single choice] *

○No income

○Less than 2,000

○2,000-2,999

○3,000-4,999

○5,000-9,999

○More than 10,000

Part 2. Sexual behavior in the past 6 month

2_1 How many male sexual partners did you have in the past 6 months? [fill in the blank] *

____

2_2 What was your frequency of condom use during sex with men in the last 6 months? [single choice] *

○Never

○Occasional

○Every time

○No sexual activity

2_3 Did you have sex with females in the past 6 months? [single choice] *

○Yes

○No

2_4 How often did you drink alcohol during or before sexual intercourse in the past 6 months? [single choice] *

○Never

○Occasional

○Often

○Always

2_5 Is there any substance you used during sexual intercourse in the past 6 months? [multiple choice] *

□None

□Rush

□"Zero" capsule

□Cocaine

□Methamphetamine

□Ketamine

□Marijuana

□Others

Part 3. HIV/STIs testing history

3_1 Have you ever taken an HIV test?? *

○Yes

○No

3_2 How many times have you been tested for HIV? [fill in the blank] *

____

3_3 When was your last HIV test? [fill in the blank] *

____

3_4 Which way did you take an HIV test by? [single choice] *

○CDC

○Hospital

○Self-testing

○Blood donation

○Others

3_5 What was the result of your last HIV test? *

○Negative

○Positive

○Uncertain

3_6 What is the reason you never took an HIV test? [multiple choice] *

□I am at low risk of HIV infection

□I fear to know the result of infection

□I don't know where to get tested

□I fear of blood drawing and needles

□Others

3_7 Are you using PrEP for HIV? [single choice]

○Yes

○No

3_8 Which of the following sexually transmitted diseases have you been diagnosed with? [multiple choice] *

□Gonorrhea

□Syphilis

□Condyloma acuminatum

□Genital tract chlamydia trachomatis infection

□Herpes progenitalis

□None

□Others

Part 4. HPV-related knowledge and vaccine acceptability

4_1 Which is the main transmission route of HPV among men? [single choice] *

○Sexual contact

○Toilet or public bath

○I don't know

4_2 Which type of cancer is caused by HPV among women? [single choice] *

○Breast cancer

○Cervical cancer

○I don't know

4_3 Which type of cancer is caused by HPV among men? [single choice] *

○Anal or penile cancer

○Lung cancer

○I don't know

4_4 Which is the main method for HPV testing in men? [single choice] *

○Blood detection

○Swab the anus or urethra

○I don't know

4_5 Could HPV cause condyloma acuminata in both men and women? [single choice] *

○Yes

○No

○I don’t know

4_6 Have you heard of the HPV vaccine? [single choice] *

○Yes

○No

4_7 Would you receive the HPV vaccine if it was free? [single choice] *

○Yes

○No

○I don’t know

The questionnaire is over. Thank you for your participation.
