# Supplementary material for: High Human Papillomavirus Vaccine Acceptability and Cost-Effectiveness of the Chinese 2-Valent Vaccine Among Men Who Have Sex With Men: A Cross-Sectional Study in Shenyang, China
Source: Front Med (Lausanne). 2021 Nov 19;8:763564. doi: 10.3389/fmed.2021.763564 (PMC8639684; doi:10.3389/fmed.2021.763564)
Supplement: Supplementary file 4 [file Data_Sheet_4.PDF]

HPV-FRAME Reporting Standards Checklist

HPV-FRAME core reporting standards

**a) Inputs**	**Reported by age? (Y/N)**	**Report by sex (F-only, M-only or both)?**	**Comments**
The target population for intervention	Y	M-only	MSM, aged 9 − 14 years
Sexual behaviour	Y	M-only	
Cohort examined for evaluation/time horizon	N	N	
Quality of life assumptions	Y	M-only	We assumed a loss of quality-adjusted life year from anal cancer according to a previous study.
Calibration	N	N	
Validation (where possible)	N	N	
Costs	Y	M-only	2020 US dollars; costs of detection, vaccination and treatment were discounted at a discount rate of 3% to account for time preferences.
**b) Outputs**	**Reported by age? (Y/N)**	**Report by sex (F-only, M-only or both)?**	**Report as calibration or validation target? (Y/N)**
Cancer incidence, mortality, life years, QALYs/DALYs (as appropriate)	N	N	N
HPV prevalence, pre-intervention	Y	M-only	N
CIN2 detected	N	N	N
Sensitivity analysis of key inputs	Y	M-only	N
Incremental cost-effectiveness ratios and costs saved	Y	Y	N

CIN: cervical intraepithelial neoplasia; F: female; M: male; Y: yes; N: no.

The citation for the HPV-FRAME reporting standards is: Canfell K, Kim JJ, Kulasingam S, Berkhof J, Barnabas R, Bogaards JA, Campos N, Jennett C, Sharma M, Simms KT et al: HPV-FRAME: A consensus statement and quality framework for modelled evaluations of HPV-related cancer control. Papillomavirus research (Amsterdam, Netherlands) 2019, 8:100184.
