# Supplementary material for: High Human Papillomavirus Vaccine Acceptability and Cost-Effectiveness of the Chinese 2-Valent Vaccine Among Men Who Have Sex With Men: A Cross-Sectional Study in Shenyang, China
Source: Front Med (Lausanne). 2021 Nov 19;8:763564. doi: 10.3389/fmed.2021.763564 (PMC8639684; doi:10.3389/fmed.2021.763564)
Supplement: Supplementary file 5 [file Table_1.DOCX]

Table S1. Cost-effectiveness evaluation of different HPV vaccination scenarios on potential anal cancer, based on 203 MSM samples

| Scenarios | No. | Estimated anal cancer patients | HPV testing cost ($) | Vaccine cost ($) | Storage and transportation cost ($) | Staff service cost ($) | Direct medical cost ($) | Direct medical cost adverted ($) | Lost QYALs (years) | Gained QYALs (year) | ICER ($ per QALY gained) |
| --- | --- | --- | --- | --- | --- | --- | --- | --- | --- | --- | --- |
| **2-valent (Cecolin^®^) vaccine** | | |  |  |  |  |  |  |  | 4.43 | 4411 |
| Willing to vaccinated | 121 | 0.9895 | 6594 | 18595 | 452 | 791 | 8089 | 6876 | 1.25 |  |  |
| Other | 82 | 0.6737 | 0 | 0 | 0 | 0 | 5507 | 0 | 5.69 |  |  |
| **2-valent (Cervarix^®^) vaccine** | | |  |  |  |  |  |  |  | 4.43 | 7929 |
| Willing to vaccinated | 121 | 0.9895 | 6594 | 34194 | 452 | 791 | 8089 | 6876 | 1.25 |  |  |
| Other | 82 | 0.6737 | 0 | 0 | 0 | 0 | 5507 | 0 | 5.69 |  |  |
| **4-valent (Gardasil^®^) vaccine** | | |  |  |  |  |  |  |  | 4.43 | 10705 |
| Willing to vaccinated | 121 | 0.9895 | 6594 | 46499 | 452 | 791 | 8089 | 6876 | 1.25 |  |  |
| Other | 82 | 0.6737 | 0 | 0 | 0 | 0 | 5507 | 0 | 5.69 |  |  |
| **9-valent (Gardasil 9^®^) vaccine** | | |  |  |  |  |  |  |  | 5.27 | 14217 |
| Willing to vaccinated | 121 | 0.9895 | 6594 | 74749 | 452 | 791 | 8089 | 7685 | 0.42 |  |  |
| Other | 82 | 0.6737 | 0 | 0 | 0 | 0 | 5507 | 0 | 5.69 |  |  |

Note: QALYs, quality-adjusted life-years; ICER, incremental cost-effectiveness ratio; Anal cancer incidence among MSM was set as 19 [10-36] per 100,000-person year (1). Direct medical cost per person with anal cancer (52500, [5,000-100,000]) was set according to the data reported by the oncology department of The First Affiliated Hospital of China Medical University. Data on the lost QALYs per person with anal cancer (8.44, [4.26-9.79]) was based on the published parameters (2). The numerator of the ICER was calculated as the projected extra costs from vaccination minus the projected increase in averted direct medical costs. The denominator was the projected gain in the number of QALYs. The calculation of the ICER can be expressed as$ICER=\frac{\left( V_{I}-V_{C} \right)-\left( A_{I}-A_{C} \right)}{\left( Q_{I}-Q_{C} \right)}$.

Table S2. Sensitivity analyses: incremental cost-effectiveness ratios (ICER) in different HPV vaccination scenarios when varying one or more parameter values.

|  | ICER ($ per QALY gained) | | | |
| --- | --- | --- | --- | --- |
| Parameter varied | 2-valent (Cecolin^®^) | 2-valent (Cervarix^®^) | 4-valent (Gardasil^®^) | 9-valent (Gardasil 9^®^) |
| None (base case) | 4411 | 7929 | 10705 | 14217 |
| One-way sensitivity analyses | | | | |
| HPV anal cancer incidence, per 100,000 person years | | | | |
| 36 | 2328 | 4185 | 5602 | 7503 |
| 10 | 8381 | 15066 | 20166 | 27011 |
| Lost QALYs, year | | | | |
| 9.79 | 3803 | 6836 | 9150 | 12256 |
| 4.26 | 8739 | 15710 | 21028 | 28166 |
| Medical treatment cost, $ |  |  |  |  |
| 100,000 | 3008 | 6526 | 9302 | 12897 |
| 5,000 | 5814 | 9332 | 12108 | 15536 |
| Multi-way sensitivity analyses | | | | |
| The lowest ICER | 1369 | 2969 | 4232 | 5868 |
| The highest ICER | 21384 | 34324 | 44533 | 57140 |

Note: the lowest and highest ICER were obtained when applying the parameter value causing the lower or higher ICER in each one-way sensitivity analyses.

1. Clifford GM, Georges D, Shiels MS, Engels EA, Albuquerque A, Poynten IM, et al. A meta-analysis of anal cancer incidence by risk group: Toward a unified anal cancer risk scale. Int J Cancer. (2021) 148(1):38-47. doi: 10.1002/ijc.33185.

2. Chesson HW, Meites E, Ekwueme DU, Saraiya M, Markowitz LE: Cost-effectiveness of nonavalent HPV vaccination among males aged 22 through 26 years in the United States. Vaccine. (2018) 36(29):4362-4368. doi: 10.1016/j.vaccine.2018.04.071.
